# Behavioural Dissociation between Exogenous and Endogenous Temporal Orienting of Attention

**DOI:** 10.1371/journal.pone.0014620

**Published:** 2011-01-28

**Authors:** Gustavo Rohenkohl, Jennifer T. Coull, Anna C. Nobre

**Affiliations:** 1 Department of Experimental Psychology, University of Oxford, Oxford, United Kingdom; 2 Department of Psychiatry, Oxford Centre for Human Brain Activity (OHBA), University of Oxford, Oxford, United Kingdom; 3 Laboratoire de Neurobiologie de la Cognition, Université de Provence & CNRS, Marseille, France; University College London, United Kingdom

## Abstract

**Background:**

In the current study we compared the effects of temporal orienting of attention based on predictions carried by the intrinsic temporal structure of events (rhythm) and by instructive symbolic cues; and tested the degree of cognitive, strategic control that could be exerted over each type of temporal expectation. The experiments tested whether the distinction between *exogenous* and *endogenous* orienting made in spatial attention may extend to the temporal domain.

**Task Design and Main Results:**

In this task, a ball moved across the screen in discrete steps and disappeared temporarily under an occluding band. Participants were required to make a perceptual discrimination on the target upon its reappearance. The regularity of the *speed* (rhythmic cue) or *colour* (symbolic cue) of the moving stimulus could predict the exact time at which a target would reappear after a brief occlusion (valid trials) or provide no temporal information (neutral trials). The predictive nature of rhythmic and symbolic cues was manipulated factorially in a symmetrical and orthogonal fashion. To test for the effects of strategic control over temporal orienting based on rhythmic or symbolic cues, participants were instructed either to “*attend-to-speed*” (rhythm) or “*attend-to-colour*”. Our results indicated that both *rhythmic* and *symbolic (colour)* cues speeded reaction times in an independent fashion. However, whilst the *rhythmic* cueing effects were impervious to instruction, the effects of *symbolic* cues were contingent on the instruction to attend to colour.

**Final Conclusions:**

Taken together, our results provide evidence for the existence of qualitatively separable types of temporal orienting of attention, akin to exogenous and endogenous mechanisms.

## Introduction

Our behaviour is adapted flexibly to meet our task goals. Selective attention research has revealed that predictions about the locations or other features of relevant events can bias neural activity starting from early stages of perceptual analysis [1], [2], [3], [4], [5], [6]. It has become increasingly evident that predictions about the timing of events also play a major role in organising and optimising behaviour [7], [8]. However, compared to spatial attention, relatively little is known about the functional mechanisms by which temporal expectations alter performance.

Like spatial orienting of attention, temporal orienting has been proposed to occur in an endogenous or exogenous fashion [9]. In spatial attention, exogenous orienting is an involuntary focusing of attention driven by an inherently salient or transient cue (also referred to as stimulus driven, automatic, bottom-up). Endogenous orienting, on the other hand, is understood as a voluntary, intentional focus of attention to a specific location, usually guided by a symbolic cue (also referred to a goal directed, controlled, top-down) [10], [11].

In spatial attention studies, the properties of exogenous and endogenous orienting are well established. Behavioural results have demonstrated that the effect of endogenous spatial orienting varies with task demands (i.e. increases with probability of cue validity), while effects of exogenous cues are independent of cue validity [12]. Studies have also suggested that exogenous cues are less susceptible to interference [12], and harder to ignore than endogenous cues [10]. Another common finding is that exogenous cues induce faster and more transient effects than endogenous orienting [13], [14].

In a recent review, Coull and Nobre [9] have suggested a distinction between endogenous and exogenous attention in the temporal domain. Exogenous temporal orienting is proposed to be generated automatically by exposure to stimuli with rhythmic or predictable temporal structure [9], [15], [16]. On the other hand, endogenous temporal orienting is proposed to be engaged in tasks that use informative, symbolic cues to direct attention in time [17], [18], [19]. Behaviourally, the effect of temporal orienting of attention has been observed both in studies using symbolic [17], [20], [21], [22], [23] as well as rhythmic cues [15], [24], [25], [26]. However, the extent to which these two different types of cues rely upon two dissociable attentional mechanisms remains unknown.

**Figure 1:**
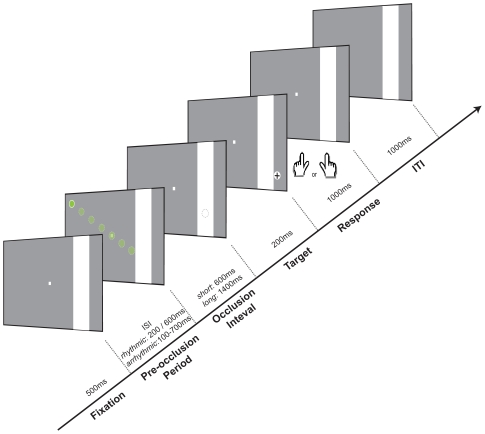
Schematic of the task. A coloured ball appears at the left side of a grey screen and moves across the screen in seven steps. After reaching the occluding band, the ball disappears behind for a single step. When the ball reappears it contains either an upright (50%) or a tilted (50%) cross. Temporal expectations could be induced by the rhythm and/or the colour of the balls preceding the occlusion.

In this experiment, we adapted the task from Doherty and colleagues [15] to test for separable effects of temporal orienting based on predictions from the intrinsic rhythmic structure of events and based on instructive symbolic cues. In our task, a ball moves across the screen of a computer monitor in discrete steps and passes ‘under’ an occluding band (see Figure 1). The participants' task is to make a speeded discrimination of a shape contained within the ball when it reappears from under the occluder. We used a factorial design to manipulate temporal expectations formed on the basis of the rhythmic temporal structure of dynamic events versus explicit symbolic cues. The temporal structure of the events was either rhythmic or arrhythmic. In rhythmic (valid) trials, the duration of each ball stimulus and of the time-step in between successive balls was constant. These parameters provided precise temporal predictions for the duration of the occlusion period, and were always valid. In arrhythmic (neutral) trials, intervals between successive balls varied randomly, providing no prediction for the duration of the occlusion period. The presence or absence of the isochronous rhythm was crossed factorially with the presence or absence of symbolic cues to predict the duration of the occlusion period. The colour of the ball acted as a symbolic cue. In trials with valid symbolic cues, the ball colour predicted the duration of the occlusion interval with 100% validity; in neutral trials the colour provided no information about the duration of the occlusion period. There were no invalid cueing conditions in the task. The design, therefore, resulted in the symmetrical and orthogonal crossing of type of temporal prediction (valid or neutral) carried by each cue type (rhythmic or symbolic).

In order to test whether expectations built from the temporal structure of events versus from symbolic cues were under cognitive control, we also manipulated task instructions in a factorial fashion. Participants were told to use the information provided by rhythm (“attend to speed”) or the colour of the balls (“attend to colour”) in different blocks. If, as we predict, temporal expectations induced by the temporal rhythm of the events are ‘exogenous’, they should not be contingent upon or affected by participants focusing on this source of information. On the other hand, if, as we predict, temporal expectations induced by symbolic cues are ‘endogenous’, they should come under cognitive control and be significantly influenced by instruction to use this source of information. Having established two separable forms of temporal orienting, we were also interested in testing whether these two sources of bias show strong interactions or whether they operate mainly independently, resulting in additive effects.

## Materials and Methods

### Participants

Eighteen right-handed participants took part voluntarily in this experiment (mean age  = 30.8 ys, 10 females). All participants had normal or corrected-to-normal vision. This study was approved by the Central University Research Ethics Committee of the University of Oxford, and all subjects provided written informed consent.

### Apparatus

The stimuli were created and presented using Presentation version 12.2 (Neurobehavioral Systems, Albany, NY). Images were displayed on a 21-inch CRT (CTX ultra screen) with a spatial resolution of 1024 by 768 pixels and a vertical refresh rate of 60 Hz, placed at 100 cm in front of the participant. Eye movements were monitored using a monocular remote video-based infrared eye tracker (ISCAN, ETL-400 system, 60 Hz). A chin rest was used to maintain a constant viewing distance and head position.

### Stimuli and Task

A schematic of the task is provided in Figure 1. The display consisted of a grey background, with a small white rectangle as the fixation point (0.29°) placed at the centre of the screen, and a white vertical occluding band (width: 3.5°) placed on the right (11.4–14.7° eccentricity). A stimulus (coloured ball - diameter:0.86°) appeared in the left hemifield (50% upper and 50% lower visual field), and moved diagonally across a computer screen in seven discrete steps (200-ms duration, see below for inter-stimulus interval) until reaching an occluding band. After a short (600-ms) or long (1400-ms) occlusion period, the target stimulus reappeared containing an upright (50%) or tilted (50%) cross, which participants were required to discriminate. Forced-choice responses were delivered using a button-press response with their right or left hand accordingly, as quickly as possible. The task was performed in the absence of eye movements. Eye tracking was performed to ensure that participants maintained central fixation throughout the active periods of the task. The factorial design had three relevant factors with two levels each: rhythmic cues (valid or neutral), symbolic cues (valid or neutral) and instruction (“attend-to-speed” or “attend-to-colour”).

Rhythmic cues were manipulated according to the duration of the inter-stimulus interval (ISI) between successive balls. In valid, rhythmic trials, the ISI was constant at either 200 ms (fast condition) or 600 ms (slow condition). Isochronous rhythms predicted the exact time at which the target would reappear after the short or long occlusion period respectively with 100% validity. (The 600-ms short occlusion is the sum of the 200-ms ISI after the ball disappears, the 200-ms duration of the invisible occluded ball, and the 200-ms ISI after the occluded ball; the 1400-ms long occlusion is the sum of the 600-ms ISI after the ball disappears, the 200-ms duration of the invisible occluded ball, and the 600-ms ISI after the occluded ball.) In arrhythmic, neutral trials, the ISI between balls varied randomly between 100–700 ms, providing no temporal prediction. Arrhythmic trials were further subdivided into three types, in which the random intervals used throughout each trial averaged to: around the short interval used in rhythmic fast trials (200 ms); the long interval used in the rhythmic slow trials (600 ms); or the intermediate interval of 400 ms. An analysis of variance (ANOVA) confirmed that performance did not differ among these different neutral trials (ps >.3), enabling the grouping of the data into one neutral condition.

Symbolic cues were manipulated according to the arbitrary association of the ball colour to one or none of the two possible occlusion intervals. In the steps leading up to the occluding band, the ball could be yellow, green or blue. Two colours predicted the duration of the occlusion with 100% validity, each signifying either the short or the long occlusion period. The third colour acted as a neutral cue, providing no specific information (50% short or long occlusion). Assignment of colour to the three cue types (valid short, valid long, neutral) was counterbalanced across participants.

The crossing of the rhythmic and symbolic cues led to four types of trials with valid or neutral, rhythmic or symbolic cues at each of the two foreperiod intervals: Valid Rhythmic and Valid Symbolic; Valid Rhythmic and Neutral Symbolic; Neutral Rhythmic and Valid Symbolic; Neutral Rhythmic and Neutral Symbolic. However, because of the effects of the passage of time, only data from the short occlusion period offer a pure measure of temporal orienting. At the short interval, the probability of a target appearing after a valid-rhythm or valid-colour trial is 1.0; whereas the probability of a target appearing after a neutral rhythm of neutral-colour is 0.5. In our task, once a target fails to appear at the short interval, its probability to appear at the long interval becomes 1.0 in all cases, regardless of whether the rhythmic or symbolic cue is valid or neutral (see [8], [27]). Consequently, at this later time period, effects of temporal orienting may be contaminated by other effects, such as re-orienting of attention [28] and foreperiod [29]. Therefore, to isolate pure effects of temporal expectation driven by rhythmic versus symbolic cues, and to rule out contamination by other sources of unwanted temporal variance, only data from the short-occlusion period were relevant.

In order to test the sensitivity of orienting based on rhythmic or symbolic cues to strategic, endogenous factors, the instructions given to the participants were also manipulated. Each block started with a screen instructing the participant to “attend to *speed*” (temporal expectations established by *rhythmic* cues) or to “attend to *colour*” (temporal expectations established by *symbolic* cues).

All participants performed a training session with 72 trials to learn the association between the cues and the occlusion period. They then proceeded to complete 10 experimental blocks of 48 trials each. Blocks with the two different instructions (“speed” or “colour”) were presented in an interleaved fashion, and the order was counterbalanced across participants. At the beginning of each block, six additional trials acted as a brief practise period to facilitate participants' engagement with the instructed condition. In total, there were 30 trials in each of the experimental conditions of interest available for analysis, based on the factors of instruction (speed, colour), rhythm (valid, neutral), symbolic cues (valid, neutral), and occlusion period (short, long). Within each block, the presentation order of different trial types was fully randomised. Trials with errors were eliminated from the analysis of reaction times (RTs).

## Results

To analyse the behavioural consequences of expectations driven by *rhythmic* versus *symbolic* temporal cues under different task instructions, RTs from correct responses were submitted to a repeated-measures analysis of variance (ANOVA), testing the three factors of instruction (speed vs. colour) *rhythmic* cues (valid vs. neutral) and *symbolic* cues (valid vs. neutral). As explained in the methods, only the data from the short-occlusion period provide a pure measure of the effects of temporal expectation.

**Figure 2:**
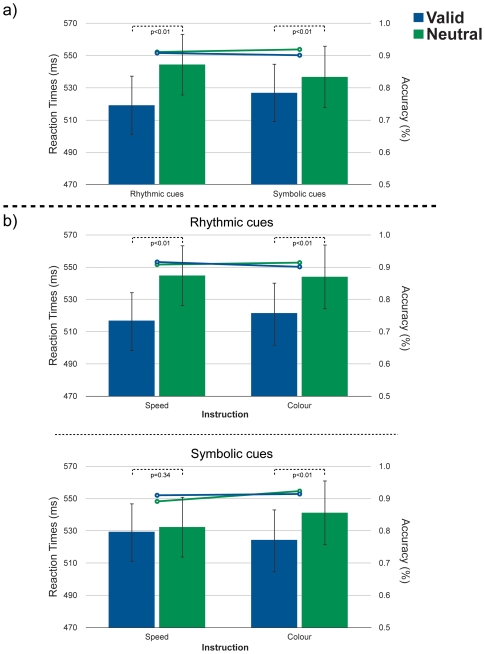
Reaction times results. *(* ***a*** *)* Main effect of temporal *rhythms* and *symbolic* cues *(b)* Effects of temporal rhythms and predictive cues broken down by task instructions. The bars represent reaction time values left y-axis), and accuracy data is represented by the lines (right y-axis). Error bars represent standard errors of the means (SE).

Performance accuracy was high (average 90.4%) and did not differentiate between conditions (lines in Figure 2), revealing no main effects or interactions between rhythmic or symbolic cueing [Fs(1, 17) <4.08, ps>.05]. In line with previous temporal orienting studies, RT values provided a sensitive measure of the effects of temporal expectations [30]. The analysis revealed main effects of *rhythmic* [F (1, 17)  = 36.23, p<.001] as well as of *symbolic* cues [F(1, 17) = 8.80, p = .009] (Figure 2a). For both cue types, valid temporal predictions speeded reaction times when compared to neutral conditions. However, whilst the effect of the temporal *rhythmic* cues was independent of instruction (main effect of rhythm), there was an interaction between *symbolic* cues and instruction [F(1, 17) = 7.03, p = .017] (Figure 2b). *Post hoc* subsidiary analyses with *Bonferroni* adjustment for multiple comparisons demonstrated that the validity effect of the *symbolic* cues was only observed if associated with the “*attend-to-colour*” instruction [F(1, 17) = 10.50, p = .005]. There were no significant effects involving the interaction between temporal expectations driven by rhythms and by symbolic cues [Fs(1, 17) <.36, ps>.55].

As expected, no differences were observed after the long occlusion period where the conditional probability of target events was equated across all trial types [Fs (1, 17) <2.23, p's>.10].

## Discussion

By using a factorial design to manipulate temporal expectations based on rhythms and symbolic cues in an orthogonal and symmetrical fashion, we provide direct evidence for the existence of qualitatively separable means of orienting of attention in time, which may be analogous to exogenous and endogenous mechanisms established in spatial orienting [9].

Similar to its spatial counterpart [12], [31], [32], [33], [34], ‘exogenous’ temporal orienting is triggered by stimulus properties and proceeds automatically. Our results confirm that the isochronous temporal rhythm of events automatically facilitates performance, regardless of whether participants strategically focus on the temporal structure of the events. This exogenous orienting appears to generate temporal expectation incidentally and automatically, allowing participants to anticipate the moment of target appearance and facilitating the perceptual discrimination process. On the other hand, symbolic cues were only effective if participants focused strategically on the information provided by the cues and voluntarily oriented their attention to the time predicted by the cue. This ‘endogenous’ type of orienting was therefore was goal-directed and strictly under top-down control.

Our results further suggested that exogenous and endogenous orienting reflect two sources of top-down biases acting upon the information-processing stream. The lack of interaction between temporal predictions carried by rhythms versus symbolic cues support the interpretation of two independent and additive mechanisms of temporal attention, at least at the processing stages that determine reaction-time performance. Further experimentation testing the effects of exogenous and endogenous effects on different behavioural measures and in different task contexts will be required to verify the generalisability of the current findings (c.f., [35], [36]). It will also be of great interest to test whether more complex, non-isochronous predictable rhythms are similarly able to induce exogenous temporal orient to facilitate performance, or whether, as the rhythms become more complex, it becomes necessary to engage voluntary, top-down mechanisms in order to benefit from learned temporal predictions [37], [38].

Our findings support Coull and Nobre's [9] proposal for a dissociation between automatic (exogenous) and goal-directed (endogenous) temporal orienting. We have also introduced a novel paradigm that allows the study of exogenous and endogenous temporal attention independently, on a trial-by-trial basis, and without requiring extensive training. Future studies are necessary to investigate the neural substrates underlying these two different orienting mechanisms of temporal attention.
